# Complete genome sequence of *Arthrobacter* sp. KFRI-F3372, a strain isolated from Korean soybean paste *Doenjang*

**DOI:** 10.1128/MRA.00588-23

**Published:** 2023-11-15

**Authors:** Sung-Hun Yi, Yu-Kyoung Park

**Affiliations:** 1 Traditional Food Research Group, Korea Food Research Institute, Wanju-gun, South Korea; University of Delaware College of Engineering, Newark, Delaware, USA

**Keywords:** *Arthrobacter*, *Doenjang*, genomes, Pacbio sequencing

## Abstract

*Arthrobacter* sp. KFRI-F3372 is a Gram-positive bacterium with a high G + C content of 65.7%, which was isolated from *Doenjang*, a traditional Korean fermented soybean paste. In this report, we introduce the complete genome sequence of *Arthrobacter* sp. KFRI-F3372.

## ANNOUNCEMENT

**Fig 1:**
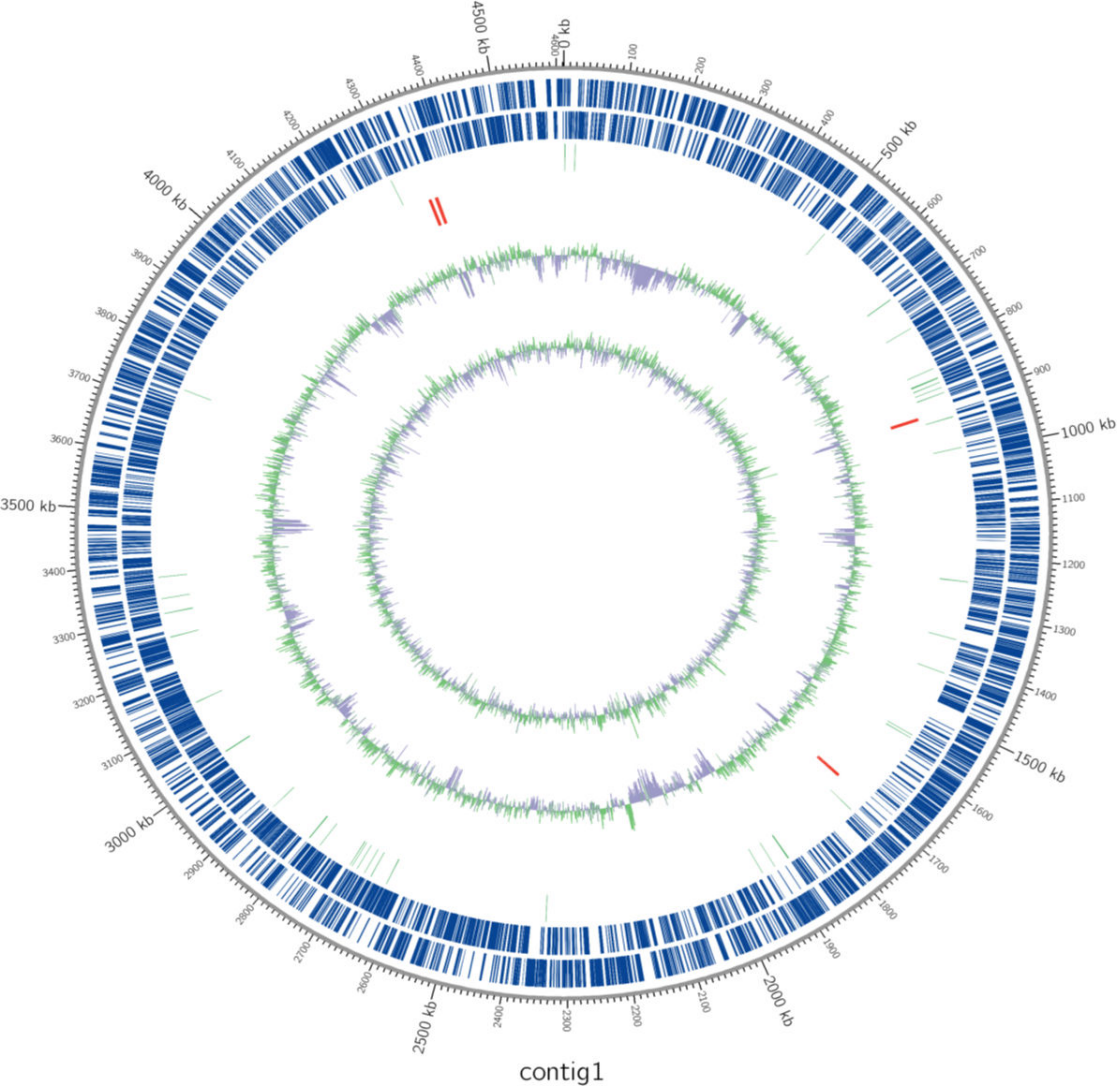
Genome map of the *Arthrobacter* sp. KFRI-F3372 circular chromosomal DNA sequence. Circular map was drawn by applying contig’s annotation result. Marked characteristics are shown from outside to the center; coding sequences (CDS) on forward strand, coding sequences on reverse strand, tRNA, rRNA, GC content, and GC skew. Regions of forward and reverse CDS are marked in blue, and region that is not CDS is described as blank. Region of tRNA is marked in light green. Region of rRNA is marked in red. Region that has a higher value of GC percentage than average is described in exterior light green peak. Otherwise, it is described in interior lavender peak. The height of the peak describes the difference from the average GC percentage. According to the formula, (G − C)/(G + C), positive value shows that G is dominant while negative value shows that C is dominant. The exterior light green peak describes the region that has higher G content while interior lavender peak describes the region that has higher C content.

*Arthrobacter* species are mainly isolated from soil and contribute to biochemical cycling and pollutant removal (1). They have been reported to be isolated from various environments (2–8). The genus *Arthrobacter* is a G(+) bacteria that can grow in various environments (9, 10). *Arthrobacter sp*. KFRI-F3372 was first isolated from a soybean paste diluted 1,000 times with physiological saline, collected from a farm in Gangwon Province, South Korea (38.123083°N, 127.802353°E) in 2006, and preserved in a freeze-dried form. Purely isolated *Arthrobacter* sp. KFRI-F3372 was grown on tryptic soy agar (BD Difco, USA), and the bacterial genomic DNA was extracted using a Maxwell Prokaryote/Eukaryote SEV DNA Purification Kit (Promega, USA) for long-read sequencing according to the manufacturer’s protocols. The genomic DNA was sheared using a g-TUBE (Covaris, USA) and purified using AMPurePB magnetic beads (Beckman Coulter Inc., USA). A total of 10 µL of the size-selected library (20 kbp) was prepared with the SMRTbell Experss Template Perp Kit 2.0. SMRTbell templates were annealed using Sequel Binding and Internal Ctrl Kit 3.0. The Sequel Sequencing Kit 3.0 and Single-Molecule, Real-Time (SMRT) cells 1M v3 Tray were used for sequencing. SMRT cells (Pacific Biosciences, USA) using 600 min movies were captured for each SMRT cell using the PacBio Sequel I (Pacific Biosciences, USA) sequencing platform by Macrogen (Seoul, Korea). Subreads generated from PacBio Sequel I system were assembled using microbial assembly application of SMRTlink 10.1.0.119588 (Hierarchical Genome Assembly Process, HGAP V4.0) (11). For error correction in long-read assembly, short-read sequencing on the Illumina HiSeq-Xten (Illumina, USA) was performed and revised using the Illumina reads to correct errors and improve accuracy with Pilon 1.21 (12). The same DNA used for PacBio sequencing was used, and it was extracted using the Maxwell Prokaryote/Eukaryote SEV DNA Purification Kit. The DNA library was prepared using the Illumina TruSeq Nano DNA library preparation protocol, in which 100 ng of high-molecular-weight genomic DNA was randomly sheared using adaptive focused acoustic technology (Covaris, USA), and the fragmented DNA was end-repaired to create 5’-phosphorylated, blunt-ended dsDNA molecules. These DNA fragments went through the addition of a single “A” base and ligation of the TruSeq DNA UD Indexing adapters. The libraries were quantified using quantitative PCR (qPCR) according to the qPCR Quantification Protocol Guide (KAPA Library Quantification kits for Illumina Sequencing platforms) and qualified using the Agilent Technologies 4200 TapeStation D1000 screentape (Agilent Technologies, USA). After quantitative qPCR, indexed libraries were subjected to short-read sequencing using the Illumina HiSeq-Xten, resulting in a total size of 1,097,248,136 bp and 7,279,220 reads. Prokka (13) v1.14.6 was used for gene prediction and basic annotation. For additional annotation, predicted protein sets were subjected to perform InterProScan (14) v5.30-69.0 and psiblast v2.4.0 (15) with EggNOG DB v4.5 (16). The PacBio Sequel I and Illumina HiSeq-Xten sequencing generated 92,760 long reads (787,295,341 bp; N_50_ 10,762 bp) and 11,334,986 read pairs (2 × 150 bp). According to the analysis using SMRT Analysis v8.0, the genome of *Arthrobacter* sp. KFRI-F3372 consists of a single circular chromosome (4,611,252 bp; G + C content, 65.7%). The contig assembly contains 4,246 total coding sequences, 50 tRNA genes, and 12 rRNA genes (Fig. 1).

## Data Availability

The complete genome sequences of *Arthrobacter* sp. KFRI-F3372 were deposited at GenBank under accession number CP125878, BioProject accession number PRJNA972333, and BioSample accession number SAMN35073509. The raw sequence data are available under SRA accession numbers SRR25113349 and SRR25113350.
